# Supplementary material for: Biosulfidogenesis Mediates Natural Attenuation in Acidic Mine Pit Lakes
Source: Microorganisms. 2020 Aug 21;8(9):1275. doi: 10.3390/microorganisms8091275 (PMC7565709; doi:10.3390/microorganisms8091275)
Supplement: Supplementary file 1 [file microorganisms-08-01275-s001.pdf]

Supplementary material

Table S1 Dissolved element concentrations measured in Filón Centro in October 2017 (top) and March 2008 (middle) as described in previous studies [3,36]; and in La Zarza in October 2017 (bottom); n.a. not analysed.

Filón Centro

October 2017

Depth	Na	K	Mg	Ca	Fe	SO_4^{2-}	SiO_2	Mn	Cu	Zn	Al	As	Be	Cd	Cr	Co	Ni	Pb	Se	Th	Tl	U	Ag	Ba	Hg	Mo	Sb	V
m	mg/l	mg/l	mg/l	mg/l	mg/l	mg/l	mg/l	mg/l	mg/l	mg/l	mg/l	µg/l	µg/l	µg/l	µg/l	µg/l	µg/l	µg/l	µg/l	µg/l	µg/l	µg/l	µg/l	µg/l	µg/l	µg/l	µg/l	µg/l
0	31.6	0.76	220	166	824	3704	137	37.7	17.6	30	166	286	8.66	71.7	40.3	1367	766	121	23	4.09	1.2	6.79	1	1.72	n.a.	< 0,8	3.59	n.a.
10	29	0.48	214	162	765	3573	130.0	36.1	16.9	28.5	157	223	8.56	70.2	40.1	1301	721	88.8	20.4	3.3	0.96	6.31	1.97	1.98	n.a.	< 0,8	2.65	n.a.
15	28.4	0.34	219	164	994	3543	133	38.6	16.1	33	191	145	8.43	89.2	43.2	1422	747	107	19.2	2.79	2.16	6.02	< 0,2	1.86	n.a.	< 0,8	3.72	n.a.
25	19.7	5.78	294	153	4159	7054	92.6	85.7	126	104	163	512	10.1	746	32	3252	1641	180	13.1	0.23	19.4	5.44	0.36	19.8	n.a.	< 0,8	6.67	n.a.
30	19.2	5.81	295	150	4023	6943	88.8	82.2	127	106	157	394	11.3	843	34	3583	1824	194	13.7	< 0,5	20.7	5.75	< 0,5	16.2	n.a.	< 2	3.57	n.a.
45	*19.6*	*17.5*	*435*	*202*	*7564*	*12339*	*30.8*	*126*	*0.165*	*151*	*28.3*	*623*	*3.96*	*5.87*	*3.09*	*5428*	*1859*	*48.7*	*7.82*	*< 0,5*	*3.89*	*2.8*	*< 0,5*	*24.3*	*n.a.*	*< 2*	*0.3*	*n.a.*

March 2008

Depth	*Na*	*K*	*Mg*	*Ca*	*Fe*	SO_4^{2-}	SiO_2	*Mn*	*Cu*	*Zn*	*Al*	*As*	*Be*	*Cd*	*Cr*	*Co*	*Ni*	*Pb*	*Se*	*Th*	*Tl*	*U*	*Ag*	*Ba*	*Hg*	*Mo*	*Sb*	*V*
m	*mg/l*	*mg/l*	*mg/l*	*mg/l*	*mg/l*	*mg/l*	*mg/l*	*mg/l*	*mg/l*	*mg/l*	*mg/l*	*µg/l*	*µg/l*	*µg/l*	*µg/l*	*µg/l*	*µg/l*	*µg/l*	*µg/l*	*µg/l*	*µg/l*	*µg/l*	*µg/l*	*µg/l*	*µg/l*	*µg/l*	*µg/l*	*µg/l*
0	*28.1*	*0.73*	*194*	*154*	*681*	*n.a.*	*n.a.*	*35.0*	*19.3*	*29.1*	*164*	*365*	*9*	*63.8*	*30.3*	*1552*	*764*	*152*	*<200*	*3.04*	*2.13*	*5.57*	*<0,8*	*<2*	*<4*	*<2*	*2.8*	*4.52*
17	*29.9*	*0.53*	*202*	*144*	*917*	*n.a.*	*n.a.*	*39.5*	*21.5*	*33.6*	*187*	*156*	*9*	*78.2*	*34.3*	*1752*	*820*	*163*	*<200*	*2.73*	*0.7*	*5.54*	*<0,8*	*<2*	*<4*	*<2*	*3.47*	*16.7*
30	*20.6*	*6.26*	*274*	*148*	*3736*	*n.a.*	*n.a.*	*71.5*	*141*	*103*	*164*	*806*	*11*	*660*	*42.9*	*2850*	*1745*	*537*	*<200*	*<2*	*13.0*	*5.43*	*<2*	*14.1*	*<10*	*<5*	*26.5*	*131*
40	*18.8*	*6.51*	*396*	*184*	*6405*	*n.a.*	*n.a.*	*108*	*40.9*	*190*	*104*	*1104*	*10*	*679*	*26.1*	*3200*	*1972*	*345*	*<200*	*<2*	*14.8*	*6.30*	*<2*	*6.26*	*<10*	*<5*	*23.8*	*65.5*

La Zarza

October 2017

Depth	*Na*	*K*	*Mg*	*Ca*	*Fe*	SO_4^{2-}	SiO_2	*Mn*	*Cu*	*Zn*	*Al*	*As*	*Be*	*Cd*	*Cr*	*Co*	*Ni*	*Pb*	*Se*	*Th*	*Tl*	*U*	*Ag*	*Ba*	*Hg*	*Mo*	*Sb*	*V*
m	*mg/l*	*mg/l*	*mg/l*	*mg/l*	*mg/l*	*mg/l*	*mg/l*	*mg/l*	*mg/l*	*mg/l*	*mg/l*	*µg/l*	*µg/l*	*µg/l*	*µg/l*	*µg/l*	*µg/l*	*µg/l*	*µg/l*	*µg/l*	*µg/l*	*µg/l*	*µg/l*	*µg/l*	*µg/l*	*µg/l*	*µg/l*	*µg/l*
0	*37.0*	*0.6*	*756*	*552*	*3,901*	*14,712*	*150*	*317*	*184*	*182*	*876*	*5,688*	*37*	*355*	*156*	*4,126*	*3,994*	*408*	*167*	*40*	*12*	*118*	*< 0,5*	*9*	*n.a.*	*3*	*18*	*177*
30	*38.9*	*8.5*	*1190*	*453*	*10,072*	*29,108*	*136*	*489*	*76.8*	*523*	*1870*	*9,878*	*59*	*1,086*	*329*	*7,295*	*6,877*	*465*	*187*	*5*	*136*	*195*	*< 1*	*14*	*n.a.*	*< 4*	*70*	*1,556*
70	*50.1*	*16.0*	*1536*	*510*	*14,334*	*37,044*	*122*	*647*	*23.2*	*624*	*2178*	*16,529*	*83*	*1,268*	*290*	*11,081*	*10,394*	*389*	*254*	*5*	*253*	*204*	*< 1*	*20*	*n.a.*	*< 4*	*74*	*1,432*

Table S2: Phosphorus as phosphate (P-PO_4^{3-}), nitrogen as nitrate (N-NO_3^-), and nitrogen as ammonium (N-NH_4^+) in the mixolimnion of Filón Centro (FC) and La Zarza (LZ) in July 2020. ND: not determined (due to interference of dissolved ferric iron concentrations).

	P-PO_4^{3-} µg/L	N-NO_3^- µg/L	N-NH_4^+ µg/L
**FC (0 m)**	146	2990	13
**LZ (0 m)**	57	ND	27

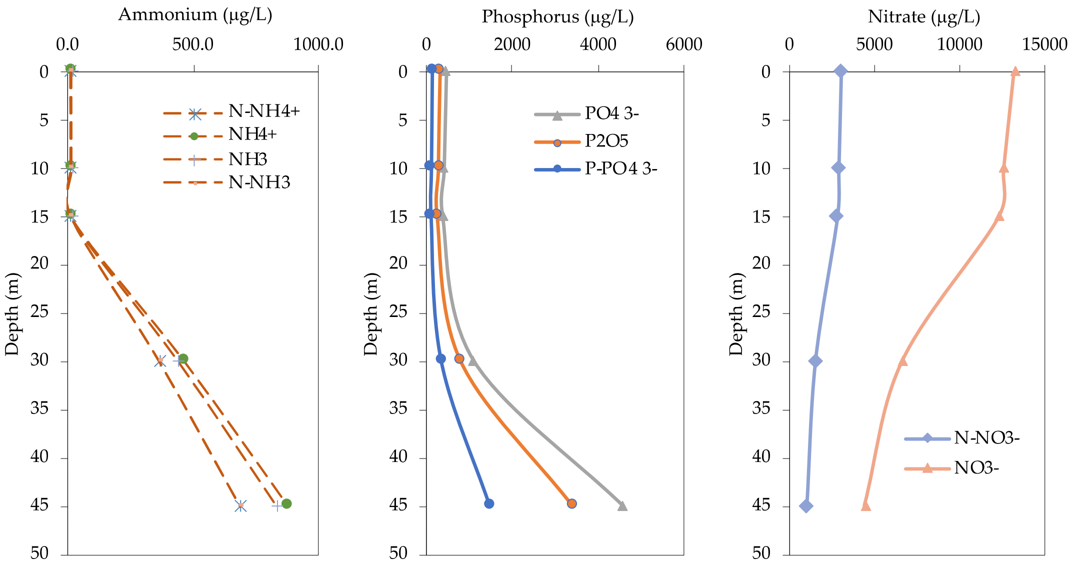

Figure S1: Nutrient profiles determined in Filón Centro in July 2020; **(a)** nitrogen as ammonium; **(b)** nitrogen as nitrate; **(c)** phosphorus as phosphate. Concentrations are given in mg/L.

Table S3. Summary of 16S rRNA amplicon sequencing data. Total number of reads remaining after quality filtering. Rarefied data (sample size 14600) was used to determine the observed ESV's per replicate, species richness (Chao1), diversity and evenness (Inverse Simpson and Simpson Index), and relative abundance of two most abundant ESV's (McNaughton's Dominance, DMN). Sample name is represented as SITE-depth-replicate

Sample Name	Filtered reads	Observed ESV's	Richness: Chao1	Diversity: Inverse-Simpson	Evenness: Simpson	Dominance: DMN
FC01r1	187063	352	363.5	4.8	0.014	0.625
FC01r2	211695	260	280.9	4.6	0.018	0.636
FC01r3	135447	254	274.3	3.9	0.015	0.686
FC15r1	181849	362	373.7	6.0	0.016	0.485
FC15r2	61905	373	400.5	6.3	0.017	0.479
FC15r3	103664	395	416.0	6.7	0.017	0.465
FC30r1	171659	483	500.4	32.8	0.068	0.145
FC30r2	121181	599	634.6	36.4	0.061	0.143
FC45r1	25576	477	503.1	4.5	0.010	0.533
FC45r2	145176	423	455.6	3.3	0.008	0.611
FC45r3	79092	377	391.2	3.6	0.010	0.600
LZ00r1	152023	216	222.7	3.2	0.015	0.647
LZ00r2	149480	232	243.7	3.2	0.014	0.624
LZ00r3	241230	259	274.1	3.3	0.013	0.612
LZ30r1	14791	786	838.4	11.8	0.015	0.344
LZ30r3	83649	693	726.1	20.0	0.029	0.241
LZ70r1	202160	538	578.1	14.7	0.027	0.314
LZ70r2	98208	320	342.5	6.7	0.021	0.513
LZ70r3	112621	772	803.0	12.0	0.016	0.326

Table S4. Distribution of PE ceramides in FC 45 and in the *M. margulisiae* isolate

	PE ceramides					
	$C_{32:0}$	$C_{33:0}$	$C_{35:0}$	$C_{35:1}$	$C_{36:0}$	$C_{37:0}$
FC 45 m	10	10	45	13	23	
M. margulisiae			43		6	51
